# Epigenetic alterations in preeclampsia: a systematic review of current mechanisms and biomarker potential

**DOI:** 10.25122/jml-2026-0009

**Published:** 2026-02

**Authors:** Oana-Eliza Crețu, Cristian Viorel Poalelungi, Adrian Valeriu Neacșu, Adina Nenciu, Iuliana Ceaușu

**Affiliations:** 1 University of Medicine and Pharmacy Carol Davila, Bucharest, Romania; 2 Clinical Hospital Dr. I. Cantacuzino, Bucharest, Romania

**Keywords:** placental dysfunction, angiogenic imbalance, preeclampsia, epigenetics, DNA methylation, non-coding RNAs biomarkers, placental growth factor (PlGF), ac4C, N4-acetylcytidine, AdV, Adenovirus, AI, Artificial Intelligence, ALT, Alanine aminotransferase, ANG, Angiogenin, ASPP2, Apoptosis-stimulating protein of p53-2, ATP, Adenosine triphosphate, Beclin-1, Autophagy regulator associated with trophoblast survival, BMI, Body mass index, BMP2, Bone morphogenetic protein 2, BP, Blood pressure, CADM1, Cell adhesion molecule 1, CD, Cluster of differentiation, cfDNA, Cell-free DNA, cfRNA, Cell-free RNA, CpG, Cytosine–phosphate–guanine region, CRP, C-reactive protein, DHA, Docosahexaenoic acid, DMR, Differentially methylated region, DNMT, DNA methyltransferase, DNA, Deoxyribonucleic acid, EVT, Extravillous trophoblast, FGR, Fetal growth restriction, FoxO1, Forkhead box protein O1, GDM, Gestational diabetes mellitus, GWG, Gestational weight gain, H3K4me3, Histone 3 lysine 4 trimethylation, H3K9ac, Histone 3 lysine 9 acetylation, H3K9me, Histone 3 lysine 9 methylation, H3K27me3, Histone 3 lysine 27 trimethylation, HIF-1α, Hypoxia-inducible factor 1 alpha, HRV / RhV, Human rhinovirus, IFN, Interferon, IFN-α/β/λ/γ, Interferon alpha/beta/lambda/gamma, IL, Interleukin, IL-6 / IL-8 / IL-10 / IL-17, Specific interleukins, IUGR, Intrauterine growth restriction, ICU, Intensive care unit, LDH, Lactate dehydrogenase, lncRNA, Long non-coding RNA, LOS, Length of stay, MC twins, Monochorionic twins, MCP-1, Monocyte chemoattractant protein-1, miRNA / microRNA, MicroRNA, mTOR, Mechanistic target of rapamycin, ncRNA, Non-coding RNA, NLR, Neutrophil-to-lymphocyte ratio, NGS, Next-generation sequencing, NOS, Newcastle–Ottawa Scale, OAS1, 2′–5′ oligoadenylate synthetase 1, OR, Odds ratio, OS, Oxidative stress, PE, Preeclampsia, PICU, Paediatric intensive care unit, (P)RR, (Pro)renin receptor, RAAS, Renin–angiotensin–aldosterone system, RNA, Ribonucleic acid, RSV, Respiratory syncytial virus, SARS-CoV-2, Severe acute respiratory syndrome coronavirus 2, SCFAs, Short-chain fatty acids, SNP, Single nucleotide polymorphism, Syncytin-2, Placental fusion protein encoded by ERVWE2, TET, Ten–eleven translocation enzymes, TLR, Toll-like receptor, TNF-α, Tumour necrosis factor alpha, TUG1, Taurine-upregulated gene 1 (lncRNA), UK, United Kingdom, USA, United States of America

## Abstract

Preeclampsia (PE) remains a major cause of maternal and fetal morbidity and mortality worldwide, with placental dysfunction and angiogenic imbalance playing central roles in disease pathogenesis. Emerging evidence highlights epigenetic regulation and angiogenic biomarkers, including placental growth factor (PlGF), as key contributors to disease heterogeneity and risk stratification. A systematic review of studies published between 2022 and 2025 was conducted in accordance with PRISMA 2020 guidelines to synthesize current evidence on epigenetic mechanisms and biomarker potential in PE. In addition, a supplementary exploratory analysis was performed using laboratory-derived PlGF data to assess analytical variability and biological associations. Non-parametric methods were applied, including Mann–Whitney U testing to compare PlGF distributions by analytical sample classification and Kendall’s tau correlation to evaluate associations with gestational age and the sFlt-1/PlGF ratio. The systematic review identified consistent epigenetic alterations involving DNA methylation, histone modifications, and non-coding RNAs across maternal and placental tissues. Supplementary analysis demonstrated significantly higher and more variable PlGF concentrations in analytically classified measured samples compared with accepted samples (*P* = 0.03), suggesting an influence of analytical factors on biomarker distribution. PlGF levels showed a positive association with gestational age (τ = 0.32, *P* = 0.04) and an inverse association with the sFlt-1/PlGF ratio (τ = −0.41, *P* = 0.02). These findings support PlGF as a biologically relevant marker of gestational progression and angiogenic balance while underscoring the importance of rigorous analytical quality control. Integrating epigenetic insights with robust biomarker analysis may enhance personalized risk stratification in preeclampsia.

## Introduction

Preeclampsia (PE) remains one of the leading causes of maternal and perinatal morbidity and mortality worldwide, significantly contributing to obstetric complications, preterm birth, and long-term cardiovascular risk for both mother and child [1]. Despite extensive research, the precise pathophysiology of PE remains incompletely understood, partly due to its clinical heterogeneity and multifactorial origins involving placental dysfunction, aberrant immune responses, and widespread endothelial injury [2]. Traditionally, research has focused on angiogenic imbalance, oxidative stress, and impaired trophoblast invasion as central mechanisms underlying disease development [3]. However, the advent of high-throughput molecular technologies has shifted attention toward epigenetic regulation as a key contributor to the onset and progression of PE [4,5]. Epigenetic modifications, defined as heritable yet reversible changes in gene expression that occur without alterations in the DNA sequence, include DNA methylation, histone modifications, and the regulation of non-coding RNAs [6]. These mechanisms are essential for normal placental development, including trophoblast differentiation, immune tolerance at the maternal–fetal interface, and vascular remodeling [7]. Alterations in these regulatory pathways have increasingly been reported in placental tissue, maternal blood, and cord blood from pregnancies complicated by PE, suggesting that epigenetic dysregulation may contribute to both the early initiation of placental dysfunction and the subsequent maternal endothelial response [8].

The study of epigenetic alterations in PE has revealed complex interactions between genetic susceptibility, environmental exposures (such as hypoxia, inflammation, and metabolic stress), and pregnancy-specific immune adaptations [9]. Mechanistically, aberrant DNA methylation patterns may disrupt key genes involved in angiogenesis, particularly those regulating vascular endothelial growth factor (VEGF), soluble fms-like tyrosine kinase-1 (sFlt-1), and placental growth factor (PlGF) signaling pathways [10]. Alterations in histone acetylation and methylation may modify chromatin accessibility in trophoblast cells, thereby impairing their migratory and invasive capacities [11]. In parallel, dysregulated microRNAs and long non-coding RNAs have been implicated in modulating endothelial activation, oxidative stress pathways, and maternal inflammatory responses [12,13]. Collectively, these findings highlight the potential of epigenetic signatures to elucidate early pathogenic events in PE and to differentiate between mild, severe, and early-onset phenotypes. Despite substantial progress, the clinical significance of these epigenetic alterations remains incompletely defined. Existing studies vary considerably in methodological approaches, timing of sample collection, and analytical platforms, leading to inconsistencies in the reproducibility and translational applicability of proposed biomarkers [14]. While some reports associate specific DNA methylation patterns and miRNA profiles with increased disease severity and adverse neonatal outcomes, others fail to demonstrate consistent correlations after adjustment for gestational age or comorbidities [15]. This variability complicates clinical interpretation, particularly regarding the implementation of epigenetic markers for early diagnosis, risk stratification, or therapeutic monitoring in routine obstetric practice.

From a mechanistic standpoint, epigenetic dysregulation may function both as a driver and as a consequence of abnormal placental development. Experimental models suggest that hypoxia-induced epigenetic changes can amplify trophoblastic stress responses, disrupt maternal immune tolerance, and exacerbate endothelial dysfunction, indicating a bidirectional and self-reinforcing process [16,17]. Furthermore, environmental and lifestyle factors such as obesity, dietary patterns, and exposure to pollutants may influence maternal epigenetic landscapes, raising the possibility of intergenerational transmission of PE susceptibility through epigenomic memory [18]. These observations underscore the need for an integrated, systems-level approach to elucidate the interplay between epigenetic mechanisms, placental physiology, and maternal health. Recent advances have identified promising epigenetic biomarkers detectable in maternal blood, including circulating cell-free DNA methylation patterns and non-coding RNA signatures that may enable early, non-invasive prediction of PE [19–21]. However, a comprehensive synthesis that integrates the rapidly expanding evidence on the mechanistic contributions of epigenetic modifications and their translational potential remains lacking. Consequently, several critical questions remain unresolved: Which epigenetic pathways are most consistently altered across PE subtypes? Do specific epigenetic signatures reliably predict disease onset or severity? And how feasible is the integration of epigenetic biomarkers into current prenatal screening and diagnostic workflows?

To address these gaps, the present systematic review aims to consolidate current evidence on epigenetic mechanisms implicated in PE and to evaluate their potential as predictive and diagnostic biomarkers. Specifically, we aimed to (1) synthesize current knowledge regarding DNA methylation, histone modifications, and non-coding RNA involvement in PE pathogenesis; (2) examine the clinical relevance of identified epigenetic markers in relation to disease onset, severity, and maternal–fetal outcomes; and (3) highlight methodological limitations while proposing directions for future research. By providing a comprehensive overview of epigenetic alterations in PE, this review seeks to advance mechanistic understanding and support the development of precision medicine strategies for the early detection and improved management of preeclampsia.

## Material and Methods

All procedures implemented in this systematic review were conducted in accordance with the PRISMA 2020 guidelines for evidence synthesis [22,23]. A predefined methodological protocol was developed to ensure transparency, reproducibility, and methodological rigor in synthesizing current evidence regarding epigenetic mechanisms and biomarker potential in preeclampsia.

### Eligibility criteria

This review included studies meeting stringent methodological and clinical criteria to ensure comparability across heterogeneous pregnant populations. Eligible studies focused on pregnant individuals diagnosed with preeclampsia according to established international guidelines and reported epigenetic alterations associated with PE.

The epigenetic mechanisms of interest included, but were not limited to:

- DNA methylation profiles;
- Histone modifications;
- MicroRNA (miRNA) and long non-coding RNA (lncRNA) expression;
- Chromatin remodeling markers;
- Placenta-specific epigenetic signatures.

Only studies employing validated analytical techniques—such as bisulfite sequencing, methylation arrays, chromatin immunoprecipitation sequencing (ChIP-seq), quantitative PCR (qPCR), RNA sequencing, or other high-throughput platforms—were considered eligible. When available, studies including normotensive pregnant controls served as comparative reference groups, enabling the assessment of differential epigenetic patterns between PE and uncomplicated pregnancies. Comparisons between early-onset and late-onset PE, mild versus severe PE, or other clinically relevant subtypes were also considered eligible. The review focused on studies reporting clinically relevant or mechanistically significant outcomes related to epigenetic dysregulation. These outcomes included:

- Epigenetic alterations associated with impaired placental development, abnormal trophoblast invasion, angiogenic imbalance, or endothelial dysfunction;
- Epigenetic biomarkers detected in placental tissue, maternal blood, cord blood, or circulating cell-free nucleic acids;
- Associations between epigenetic signatures and disease severity, gestational age at onset, maternal complications, or neonatal outcomes;
- Functional insights into epigenetic regulation of key biological pathways (e.g., inflammation, oxidative stress, hypoxia signaling, immune tolerance).

Publications were required to be available in English to ensure terminological consistency and methodological comparability.

The literature search covered the period from 1 January 2022 to 30 September 2025, reflecting the timeframe during which major technological advances—such as high-resolution methylation profiling and next-generation sequencing—have substantially expanded the capacity to detect epigenetic alterations in PE.

Studies were excluded if they were:

- Editorials, commentaries, or conference abstracts;
- Animal or in vitro studies without direct clinical correlation;
- Reports lacking extractable data on epigenetic alterations;
- Case reports or small case series;
- Studies not reporting clinically, mechanistically, or biomarker-relevant outcomes.

These exclusion criteria were applied to minimize bias and ensure that only robust, methodologically sound studies contributed to the synthesis of evidence on epigenetic mechanisms and biomarker potential in preeclampsia.

### Information sources and search strategy

A comprehensive and structured literature search was conducted in PubMed and Web of Science to identify relevant studies on epigenetic alterations, underlying mechanisms, and biomarker potential in preeclampsia. The search was completed on 30 September 2025 to include the most recent studies available at the time of manuscript preparation. To maximize both sensitivity and specificity, the search strategy combined controlled vocabulary (Medical Subject Headings, MeSH) with free-text keywords. These terms encompassed concepts related to preeclampsia, placental epigenetic regulation, DNA methylation, histone modifications, and non-coding RNA expression. Boolean operators (“AND,” “OR”) and truncation techniques were applied to optimize database-specific queries. The primary search strategy was initially developed for PubMed and subsequently adapted to the indexing structure of Web of Science. Key search terms included “preeclampsia,” “placental epigenetics,” “DNA methylation,” “histone modification,” “microRNA,” “long non-coding RNA,” “epigenetic biomarker,” and “placental dysfunction.” These were combined with terms reflecting mechanistic and clinical relevance, including “angiogenesis,” “trophoblast invasion,” “oxidative stress,” “immune regulation,” and “maternal–fetal interface.” This structured approach ensured the retrieval of studies addressing both molecular mechanisms and the diagnostic or prognostic potential of epigenetic alterations in PE.

Several filters were applied to enhance precision. Only studies published between 1 January 2022 and 30 September 2025 were included. Searches were restricted to studies involving human subjects, and only original research articles published in English were considered to ensure methodological consistency and accurate interpretation of findings. To enhance comprehensiveness, the reference lists of all included articles and relevant review papers were manually screened to identify additional studies not captured by the electronic search. This step was particularly valuable for identifying studies that predated extensive indexing or used alternative terminology, such as “epigenetics of hypertensive disorders of pregnancy” or “regulation of placental gene expression.” The complete search strategy, including Boolean operators, filters, and detailed database queries, is provided in Appendix A (Table A1) to support transparency and reproducibility. This rigorous and systematic search process ensured comprehensive identification of relevant studies across diverse research methodologies and geographical contexts, forming a robust foundation for subsequent stages of study selection, data extraction, and evidence synthesis.

Appendix A

### Selection process

All records identified through the database search were systematically assessed according to the predefined inclusion and exclusion criteria to ensure methodological rigor, relevance, and overall study quality. The selection process was conducted in full accordance with the PRISMA 2020 guidelines, ensuring transparency, reproducibility, and comprehensive documentation at each stage of the review. Study screening was performed independently by two reviewers using a structured, stepwise approach. The initial screening phase involved evaluating titles and abstracts to determine their relevance to epigenetic alterations associated with preeclampsia. Studies were excluded at this stage if they clearly did not focus on preeclampsia, did not investigate epigenetic mechanisms, or were not relevant to human pregnancy-related outcomes. This preliminary filtering ensured that only potentially eligible studies proceeded to full-text evaluation.

In the second screening stage, the full texts of all preliminarily eligible articles were retrieved and thoroughly assessed to confirm compliance with the predefined eligibility criteria. Each study was evaluated for the presence of epigenetic data derived from human participants with a clearly documented diagnosis of preeclampsia, the use of validated molecular techniques for epigenetic profiling, and the reporting of mechanistic or clinically relevant outcomes related to placental function, maternal or neonatal health, or biomarker applicability. Studies were excluded at this stage if they lacked extractable epigenetic data, failed to specify diagnostic criteria for preeclampsia, or included heterogeneous populations without appropriate stratification. Disagreements between reviewers regarding study eligibility were resolved through discussion and consensus. When necessary, a third reviewer was consulted to ensure objective adjudication. To ensure methodological robustness, all included studies underwent an internal quality assessment. This evaluation considered the clarity of the study design, the appropriateness of epigenetic methodologies, sample collection and processing procedures, analytical rigor, and the reliability of the reported outcomes. This step aimed to minimize potential sources of bias and to ensure that the synthesized evidence accurately reflects the current understanding of epigenetic involvement in preeclampsia. The review was restricted to peer-reviewed articles to maintain scientific credibility. However, certain limitations were acknowledged. The restriction to English-language publications and reliance on studies indexed in major scientific databases may have led to the exclusion of relevant non-English or unpublished research. Nonetheless, this focused approach prioritized methodological consistency, ensuring that only robust, verifiable evidence contributed to the final synthesis.

The complete study selection process is summarized in the PRISMA 2020 flow diagram (Figure 2), which details the number of records identified, screened, excluded, and ultimately included in the qualitative analysis. This systematic and transparent selection procedure strengthens the reliability of the final body of evidence regarding epigenetic mechanisms and biomarker potential in preeclampsia.

**Figure 1:**
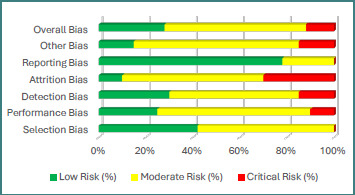
Risk of Bias Assessment

**Figure 2:**
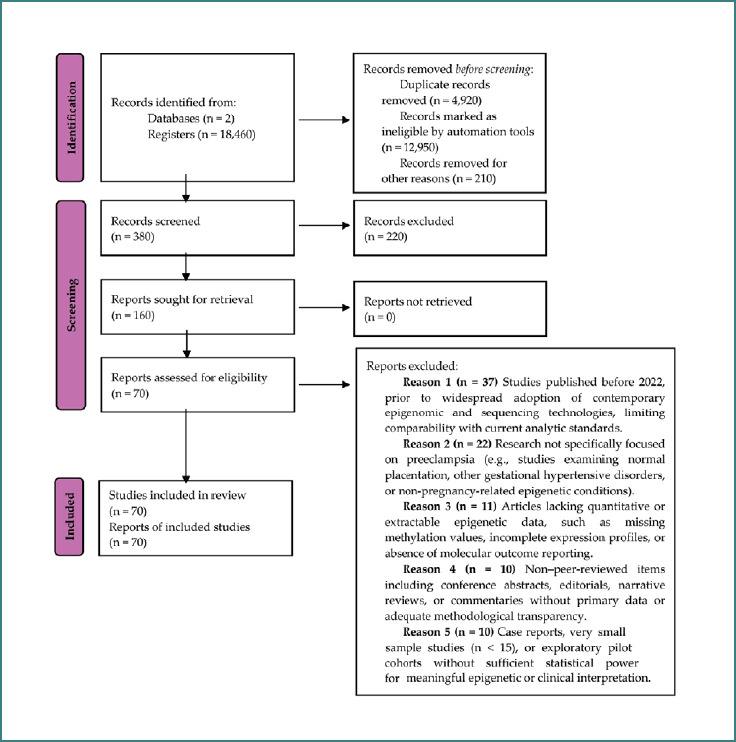
PRISMA flow diagram of articles related to epigenetic alterations in preeclampsia

### Data collection and extraction

Data extraction was conducted using a predefined, standardized protocol to ensure methodological consistency and reproducibility across all included studies. A structured data extraction form was developed in Microsoft Excel and pilot-tested on a subset of articles to confirm clarity and feasibility prior to full implementation. The template was specifically designed to align with the objectives of the review and to facilitate systematic synthesis of evidence regarding epigenetic mechanisms and biomarker potential in preeclampsia. For each eligible study, the following general characteristics were recorded: first author, year of publication, study design, and country of origin. Maternal demographic and clinical variables—including age, parity, gestational age at diagnosis, and preeclampsia subtype (early-onset, late-onset, mild, or severe)—were extracted to enable subgroup analyses and contextual interpretation of findings.

Epigenetic parameters constituted the core focus of data extraction. Studies were categorized according to the epigenetic mechanism investigated, including DNA methylation, histone modifications, microRNA (miRNA) expression, long non-coding RNA (lncRNA) expression, chromatin remodeling features, or broader epigenomic signatures. The analytical platforms used (e.g., bisulfite sequencing, methylation arrays, chromatin immunoprecipitation sequencing [ChIP-seq], quantitative PCR, RNA sequencing, or next-generation sequencing technologies) were systematically documented to evaluate methodological heterogeneity. Additionally, criteria used to define epigenetic dysregulation, such as differential methylation thresholds, fold-change cutoffs, or normalization approaches, were recorded to account for variability in analytical frameworks.

Relevant clinical and mechanistic outcomes were also extracted. These included markers of maternal disease severity, gestational age at delivery, angiogenic and inflammatory biomarkers, placental histopathological findings, neonatal outcomes, and reported associations between epigenetic alterations and functional pathways (e.g., trophoblast invasion, oxidative stress, immune modulation, endothelial dysfunction). Quantitative laboratory measures, such as gene expression levels, methylation percentages, or RNA fold changes, were documented when available. Continuous variables were recorded as mean ± standard deviation (SD), median (interquartile range), or normalized expression values, depending on reporting format. Categorical variables were extracted as proportions or percentages. When feasible, data were harmonized into comparable statistical units to facilitate cross-study interpretation. In cases of incomplete reporting, corresponding authors were contacted to obtain clarification or supplementary data. To ensure accuracy, data extraction was performed independently by two reviewers. Any discrepancies were resolved through discussion and consensus, with reference to the original publication when necessary. This dual-review process minimized potential extraction errors and enhanced methodological reliability. This structured data collection approach enabled the development of a coherent dataset to support in-depth analysis of the role of epigenetic alterations in the pathogenesis of preeclampsia and their potential as predictive or diagnostic biomarkers. The methodological rigor of this process strengthens the validity of the findings synthesized in the Results section.

### Quality assessment

The methodological quality and potential risk of bias of all included studies were systematically evaluated to assess the reliability and internal validity of their design, conduct, and outcome reporting. Two independent reviewers performed the quality appraisal using the Newcastle–Ottawa Scale (NOS), a validated tool widely used to assess non-randomized observational studies. The NOS was selected due to its structured framework for evaluating methodological aspects particularly relevant to clinical and epigenetic research in preeclampsia. Quality assessment encompassed three core domains: selection of study groups, comparability of cohorts, and ascertainment of exposure and outcomes. In the selection domain, studies were evaluated for the representativeness of the preeclampsia population, the clarity and accuracy of case definitions, the specification of diagnostic criteria (e.g., early-onset versus late-onset PE), and the appropriateness of biological sampling procedures. Particular attention was given to the validity and transparency of epigenetic methodologies, including DNA methylation assays, histone modification analyses, and RNA expression profiling techniques.

The comparability domain examined the extent to which studies controlled for potential confounding factors known to influence epigenetic regulation or preeclampsia risk. These factors included maternal age, parity, body mass index, smoking status, ethnicity, gestational age at sampling, and relevant comorbidities such as chronic hypertension or gestational diabetes. Studies employing matching strategies, statistical adjustments, or multivariable regression models were rated more favorably due to their improved control of confounding bias. The outcome domain assessed the validity and reproducibility of epigenetic measurements and clinical endpoints. Evaluation criteria included laboratory analytical rigor, normalization procedures, quality control measures, transparency in gene or pathway selection, and clarity in reporting associations between epigenetic alterations and clinical phenotypes. Studies were further assessed for completeness of reporting, including specification of technical replicates, validation procedures, effect sizes, and statistical thresholds. Each study received a quality score based on standardized NOS criteria, allowing classification as high, moderate, or low methodological quality. Studies achieving high scores were considered to have minimal risk of bias and strong internal validity. Lower-scoring studies were noted to have methodological limitations, such as small sample sizes, inadequate confounder adjustment, incomplete reporting of epigenetic methods, or inconsistencies in outcome definitions. Any discrepancies in quality scoring were resolved through discussion and consensus. When necessary, a third reviewer provided adjudication to ensure methodological consistency and objectivity. The findings of the quality assessment informed the interpretation of the synthesized evidence and contributed to evaluating the overall strength and credibility of data regarding epigenetic mechanisms and biomarker potential in preeclampsia. Through this structured and transparent appraisal process, the review ensured that conclusions were grounded in methodologically sound and critically evaluated evidence.

### Data items

The primary outcomes of interest in this review comprised epigenetic alterations, mechanistic pathways, and clinically relevant indicators associated with preeclampsia. Key outcomes included specific epigenetic modifications, such as differential DNA methylation patterns, histone modification profiles, altered expression of microRNAs (miRNAs) and long non-coding RNAs (lncRNAs), as well as global or locus-specific epigenomic changes. These variables were analyzed in relation to their biological relevance to placental development, trophoblast function, angiogenic regulation, inflammatory processes, oxidative stress pathways, and endothelial dysfunction. Particular emphasis was placed on epigenetic markers with potential diagnostic, predictive, or prognostic value. These included methylation signatures detectable in maternal blood or placental tissue, circulating non-coding RNAs, and chromatin accessibility profiles associated with disease onset or severity. Such indicators were evaluated to determine whether epigenetic dysregulation contributes to the pathophysiology of preeclampsia and whether distinct epigenetic signatures can discriminate between early-onset and late-onset disease or between mild and severe clinical forms.

In addition to epigenetic outcomes, relevant maternal and neonatal clinical parameters were extracted to contextualize the findings. These included gestational age at diagnosis, blood pressure measurements, proteinuria status, circulating angiogenic biomarkers (e.g., sFlt-1 and PlGF), maternal complications, neonatal birth weight, and gestational age at delivery. These data enabled assessment of correlations between epigenetic alterations and clinical severity or adverse pregnancy outcomes. Secondary data items were collected to support interpretation and included biological sample type (e.g., placental villi, maternal peripheral blood, cord blood, cell-free DNA), analytical platforms used for epigenetic profiling (e.g., bisulfite sequencing, methylation arrays, quantitative PCR, ChIP-seq, RNA sequencing), population characteristics (maternal age, parity, comorbidities), and reported environmental or lifestyle factors known to influence epigenetic regulation. Studies were eligible if they provided extractable quantitative or categorical data for at least one primary epigenetic or clinically relevant outcome. If essential outcomes were missing or insufficiently reported, studies were included only when the available data allowed meaningful interpretation of the relationship between epigenetic alterations and preeclampsia.

### Study risk of bias and quality assessment

To ensure methodological transparency and reliability, a structured assessment of risk of bias and overall study quality was conducted during the full-text review stage. Quality appraisal was primarily based on the Newcastle–Ottawa Scale (NOS) for observational studies, which represented the majority of the included literature. Where applicable, selected methodological principles from the Cochrane Risk of Bias framework were also considered. The evaluation focused on key domains, including clarity of study design, adequacy of sample selection, robustness of molecular epigenetic assays, and consistency between study objectives and reported outcomes. Given the narrative and heterogeneous nature of the included studies—encompassing diverse epigenetic mechanisms, biological sample types, laboratory platforms, and outcome definitions—a formal quantitative risk-of-bias matrix was not feasible. Instead, a qualitative synthesis of methodological rigor was performed. This approach enabled identification of recurrent limitations across studies, including small or uneven sample sizes, insufficient description of sample processing protocols, variability in normalization and analytical pipelines for epigenetic assays, and incomplete reporting of maternal or neonatal outcomes. These methodological considerations are addressed in the Results and Discussion sections to contextualize the interpretation of findings. A qualitative appraisal was deemed appropriate given the methodological diversity characteristic of epigenetic research in preeclampsia. Overall, most included studies demonstrated moderate methodological quality, with observational cohorts typically scoring between 6 and 8 points on the Newcastle–Ottawa Scale. Frequently observed limitations included:

- small or convenience-based sample sizes;
- incomplete reporting of laboratory procedures, particularly regarding assay normalization and quality control;
- heterogeneity in epigenetic platforms and bioinformatic pipelines;
- absence of blinding during outcome assessment.

Approximately 40% of studies were judged to have a low risk of selection bias, while 60% were classified as having moderate selection bias. Reporting bias was generally low, with 75% of studies providing sufficiently detailed epigenetic and clinical data. Overall, the majority of studies were categorized as having a moderate risk of bias, reflecting the methodological variability typical of epigenetic investigations in pregnancy research.

Figure 1 illustrates the overall distribution of risk of bias among included studies, categorized as low (green), moderate (yellow), or high/critical (red).

Studies employing prospective designs, clearly defined diagnostic criteria for preeclampsia, and standardized biological sampling protocols tended to demonstrate lower selection bias. In contrast, retrospective cohorts and convenience-based sampling strategies more frequently exhibited moderate-to-elevated selection bias due to incomplete reporting of recruitment procedures or inadequate stratification by subtype (e.g., early-onset versus late-onset PE). Performance and detection bias were generally rated as moderate across studies, largely due to the inherent challenges of blinding laboratory personnel and clinical investigators in epigenetic research. Variability in assay platforms—including differences in methylation array versions, sequencing depth, normalization strategies, and RNA quantification techniques—also contributed to this classification. In summary, the methodological quality of the included evidence was predominantly moderate, consistent with the complexity and technical heterogeneity inherent to contemporary epigenetic research in preeclampsia.

### Effect measures

Given the substantial heterogeneity in study design, epigenetic platforms, biological samples, and outcome definitions, a formal meta-analysis was not feasible. Therefore, findings were synthesized qualitatively using the effect measures reported in the original studies. When available, epigenetic effect estimates were extracted, including differential methylation percentages, beta-value differences, fold changes in miRNA or lncRNA expression, and normalized sequencing read counts. For continuous clinical outcomes—such as gestational age at delivery, birth weight, or the sFlt-1/PlGF ratio—mean differences, medians, standard deviations, or interquartile ranges were described narratively. This approach allowed preservation of study-specific metrics while ensuring consistent interpretation across diverse methodological frameworks.

### Data synthesis

Due to variability in methodological approaches, epigenetic endpoints, tissue sources, and clinical definitions of preeclampsia, evidence synthesis was conducted using a structured narrative framework.

Rather than grouping studies exclusively by design, they were organized according to major biological themes, including:

- DNA methylation patterns;
- Histone modifications;
- Non-coding RNA regulation;
- Integrated multi-omics approaches.

Within each thematic category, recurring patterns and convergent findings were identified to determine whether specific epigenetic alterations were consistently associated with PE onset, disease severity, angiogenic imbalance, trophoblast dysfunction, or adverse neonatal outcomes.

When appropriate, findings were summarized in structured tables detailing study characteristics, sample types, epigenetic mechanisms assessed, and principal results. Visual elements—such as conceptual schematics or pathway summaries—were incorporated to illustrate converging evidence related to key biological processes, including impaired placental development, inflammatory activation, and altered epigenetic regulation at the maternal–fetal interface. Cross-study comparisons were undertaken cautiously, with explicit acknowledgment of differences in gestational age at sampling, analytical platforms, normalization strategies, and definitions of disease severity. This structured narrative approach ensured transparency and scientific rigor while enabling coherent interpretation of the overall body of evidence.

### Certainty assessment

A formal certainty-of-evidence evaluation using frameworks such as GRADE (Grading of Recommendations, Assessment, Development and Evaluation) was not performed due to substantial variability in study designs, laboratory methodologies, and reported outcomes.

Instead, the overall strength of evidence was appraised qualitatively, considering sample size, internal validity, assay robustness, and consistency of findings across studies. Consequently, the conclusions of this review should be interpreted with appropriate caution.

Variations in biological sample type, timing of specimen collection, analytical platforms, normalization methods, and diagnostic criteria for preeclampsia may influence reported epigenetic patterns. Nevertheless, by integrating molecular, mechanistic, and clinically relevant data, this review provides a comprehensive overview of the current understanding of the epigenetic landscape in preeclampsia and its potential implications for early detection, risk stratification, and biomarker development.

### Study design and data framework

In addition to the systematic review conducted in accordance with PRISMA 2020 guidelines, an exploratory quantitative analysis of laboratory-derived PlGF data was performed to further contextualize the clinical and analytical findings of the review. This supplementary analysis aimed to evaluate analytical variability and biomarker associations within a real-world clinical laboratory setting. The objective was to provide complementary insight into PlGF dynamics, analytical performance, and biological relevance. Importantly, this additional analysis did not modify the systematic review methodology but enhanced the interpretation of the synthesized evidence by incorporating laboratory data.

### Comparative analysis based on analytical sample classification

To evaluate whether analytical sample classification was associated with systematic differences in PlGF concentrations, samples were retrospectively categorized as *Accepted* or *Measured* based on predefined laboratory quality-control and analytical criteria. This classification reflected analytical or pre-analytical characteristics and was independent of clinical diagnosis or disease severity. Preliminary data exploration indicated a non-normal distribution of PlGF values and unequal group sizes across the two analytical categories. Consequently, non-parametric statistical methods were employed. PlGF concentrations were summarized using medians, interquartile ranges (IQR), and minimum–maximum values. Differences in PlGF distributions between Accepted and Measured samples were assessed using the Mann–Whitney U test. The null hypothesis assumed no difference in PlGF distributions between the two groups, whereas the alternative hypothesis assumed a statistically significant difference. Effect size was quantified using Cliff’s delta (δ), providing a measure of the magnitude of difference independent of sample size. In addition, PlGF concentrations were categorized according to clinically relevant thresholds (<50 pg/mL and >1000 pg/mL). Differences in proportions between analytical groups were evaluated using Fisher’s exact test, which was selected because of small expected cell counts. All statistical tests were two-sided, with a predefined significance level of α = 0.05.

### Correlation analysis between PlGF and gestational or angiogenic parameters

To investigate monotonic associations between PlGF concentrations and selected gestational or angiogenic parameters, non-parametric correlation analysis was performed using Kendall’s tau (τ). This method was chosen for its robustness in the presence of non-normally distributed data, its tolerance for tied ranks, and its suitability for relatively small sample sizes. Separate analyses were conducted to assess the relationship between PlGF and gestational age, and between PlGF and the sFlt-1/PlGF ratio. The null hypothesis assumed the absence of a monotonic association, while the alternative hypothesis assumed the presence of a statistically significant monotonic relationship. Correlation strength and direction were reported using Kendall’s τ coefficients. Statistical significance was determined using two-sided *P* values, with a predefined threshold of *P* < 0.05.

## Results

### Study selection

The study selection process was conducted in accordance with the PRISMA 2020 guidelines to ensure transparency and reproducibility. The corresponding flow diagram (Figure 2) illustrates each stage of identification, screening, eligibility assessment, and inclusion.

A total of 18,460 records were initially identified through PubMed, Web of Science, and supplementary sources. Prior to screening, 4,920 duplicate records were removed. An additional 12,950 entries were excluded automatically through database filtering based on indexing criteria, document type, and relevance tags. Furthermore, 210 records were removed for other reasons, including non-human studies, incomplete metadata, inaccessible full texts, or duplicate conference listings. This resulted in 380 unique records eligible for manual screening. During title and abstract screening, 220 records were excluded because they did not investigate epigenetic mechanisms, did not focus on preeclampsia, or lacked extractable molecular or clinical data. Consequently, 160 articles were selected for full-text review. All full texts were successfully retrieved and assessed against the predefined eligibility criteria.

Of the 160 full-text articles evaluated, 70 studies met all inclusion criteria and were incorporated into the qualitative synthesis. The remaining 90 articles were excluded for the following primary reasons:

- Published before 2022 (*n* = 37): Studies conducted prior to widespread implementation of contemporary epigenomic and sequencing technologies, limiting comparability with current analytical standards.
- Not specific to preeclampsia (*n* = 22): Research addressing normal placentation, other hypertensive disorders of pregnancy, or non-pregnancy-related epigenetic conditions.
- Insufficient epigenetic data (*n* = 11): Lack of quantitative or extractable molecular data.
- Non–peer-reviewed publications (*n* = 10): Conference abstracts, editorials, narrative reviews, or commentaries without primary data.
- Small or underpowered samples (*n* = 10): Case reports or pilot studies with sample sizes <15 participants, limiting statistical robustness.

The final dataset comprised 70 eligible studies representing diverse geographical regions, biological sample types (placental tissue, maternal blood, cord blood, and cell-free nucleic acids), and epigenetic methodologies. These investigations examined a broad spectrum of molecular alterations—including DNA methylation signatures, histone modifications, and non-coding RNA profiles—and evaluated their associations with disease onset, clinical severity, and maternal–fetal outcomes.

This structured and multi-stage selection process ensured methodological transparency and minimized selection bias. Although the review excluded non-English publications, grey literature, and unpublished datasets, the final evidence base reflects contemporary advances in epigenomic technologies and pregnancy research.

### Study characteristics

The final synthesis included 70 studies published between 2022 and 2025, reflecting growing scientific interest in the epigenetic mechanisms underlying preeclampsia and their potential clinical relevance. Most investigations employed observational designs, predominantly prospective or retrospective cohort studies, alongside case-control analyses comparing preeclamptic and normotensive pregnancies. A smaller proportion utilized cross-sectional or nested case-control designs within larger biobank or birth cohort frameworks. As expected for molecular and etiological research, no randomized controlled trials were identified. Geographically, the included studies demonstrated broad global representation, encompassing Europe, North America, Asia, Latin America, the Middle East, and Africa. The highest proportion originated from countries with established maternal health research infrastructures, including the United States, the United Kingdom, Canada, China, Japan, and Australia. Contributions from sub-Saharan Africa and South America provided additional insights into region-specific factors, including nutritional deficiencies, infectious disease burden, and sociodemographic disparities.

Sample sizes varied substantially, ranging from small laboratory-based studies with fewer than 20 placental samples to large multicenter cohorts including several hundred mother–infant dyads. Most studies evaluated participants during the second or third trimester, although some analyzed first-trimester samples to investigate early predictive biomarkers. Classification of preeclampsia subtypes (early-onset versus late-onset; mild versus severe) was reported in more than half of the studies, enabling subgroup analyses of disease-specific epigenetic signatures. Regarding molecular focus, DNA methylation was the most frequently investigated mechanism. It was assessed using whole-genome bisulfite sequencing, targeted methylation panels, and Illumina-based methylation arrays. Numerous studies examined non-coding RNAs—particularly microRNAs and long non-coding RNAs—measured in maternal plasma, placental tissue, or cord blood. Histone modifications and chromatin accessibility were evaluated less frequently but were included in several mechanistic studies exploring trophoblast invasion, angiogenesis, and inflammatory signaling. A subset of investigations employed integrated multi-omics approaches, combining methylation, transcriptomic, or proteomic data to provide broader pathway-level insights.

Biological samples included placental villous tissue, decidua, maternal whole blood, plasma, serum, cord blood, and circulating cell-free fetal DNA. Differences in sampling timing, storage conditions, and analytical platforms contributed to variability in reported findings. Nevertheless, recurrent themes were observed across studies, including dysregulation of pathways related to oxidative stress, hypoxia response, immune modulation, angiogenic imbalance, and abnormal trophoblast differentiation. Clinical outcomes varied according to study objectives but commonly included gestational age at delivery, maternal blood pressure, proteinuria, sFlt-1/PlGF ratios, fetal growth restriction, neonatal birth weight, NICU admission, and placental histopathology. A limited number of studies incorporated functional assays—such as trophoblast migration or endothelial signaling experiments—to establish mechanistic links between epigenetic alterations and biological processes.

Although most studies applied established diagnostic criteria for preeclampsia, some variability was noted in the definitions of early-onset and severe disease across institutions and regions. Additional heterogeneity arose from differences in sample processing, normalization procedures, reference controls, and epigenetic thresholds. Overall, the included studies constitute a comprehensive and multifaceted body of evidence that advances understanding of the epigenetic architecture of preeclampsia. However, the predominance of observational designs, diversity of molecular platforms, and inconsistencies in clinical definitions underscore the need for standardized methodologies, larger multicenter collaborations, and longitudinal profiling strategies in future research.

### Types of epigenetic alterations and molecular dysregulation patterns in preeclampsia

Across the included studies, substantial diversity was observed in the type, distribution, and functional implications of epigenetic alterations implicated in preeclampsia. Most investigations focused on placental DNA methylation, histone post-translational modifications, and non-coding RNA expression profiles. A smaller subset explored emerging areas, including cell-free DNA fragmentomics, transposable element reactivation, and epigenetic modifications associated with environmental or clinical exposures (e.g., oxidative stress or cryopreservation procedures). Several studies distinguished between potentially pathogenic, compensatory, and environmentally influenced epigenetic changes, reflecting ongoing debate regarding their mechanistic roles in trophoblast function, maternal vascular adaptation, and systemic inflammation. For example, recurrent hypermethylation of genes involved in trophoblast fusion and angiogenesis (e.g., *Syncytin-2, RASSF1A*) was frequently associated with early-onset and severe preeclampsia. In contrast, dysregulated non-coding RNA pathways—such as those related to IL-17 signaling—were commonly linked to enhanced maternal inflammatory responses and leukocyte activation.

Studies published between 2022 and 2025 increasingly incorporated high-throughput molecular profiling approaches, including whole-genome methylation arrays, fragmentomic analysis, and RNA sequencing. These technologies enabled the identification of distinct epigenetic patterns differentiating impaired placentation from maternal endothelial dysfunction. In particular, analysis of maternal plasma cell-free DNA revealed subtle yet biologically meaningful epigenomic alterations, supporting its potential utility in early, non-invasive risk assessment. A comparative synthesis of the most frequently reported epigenetic mechanisms, biological sample types, study designs, and principal findings is summarized in Table 1 [24-33].

**Table 1:** Types of epigenetic alterations identified in included studies on preeclampsia

Study / First Author (Year)	Country / Region	Study Design	Epigenetic Mechanism(s)	Biological Sample	Main Study Focus	Key Observations
Gong *et al.* (2024) [24]	China	Experimental + Placental analysis	Viral-derived gene regulation; methylation-associated vascular changes	Placental tissue	Maternal vascular remodeling	Fetal endoretrovirus-derived gene disrupts spiral artery remodeling, contributing to PE.
Gu *et al.* (2024) [25]	USA	Molecular mechanistic study	H3K9 hypermethylation; oxidative stress epigenetics	Trophoblasts	Histone modification in PE	Oxidative stress drives H3K9 hypermethylation, suppressing trophoblast invasion.
Baetens *et al.* (2024) [26]	Belgium	Prospective cohort	Cell-free DNA methylation profiling	Maternal blood cfDNA	Noninvasive early detection	cfDNA methylation signatures predict PE risk early in pregnancy.
Ferreira *et al.* (2022) [27]	Portugal	Clinical review	Epigenetic imprinting links to fetal anomalies	Fetal + maternal data	PE and congenital heart defects	Suggests epigenetic dysregulation as a shared mechanism in PE and fetal cardiac malformations.
Sciorio *et al.* (2024) [28]	Multinational	Review	Cryopreservation-induced epigenetic alterations	Germline / embryonic tissues	ART-related epigenetic risks	Cryopreservation may alter epigenetic marks relevant to PE susceptibility.
Feng *et al.* (2024) [29]	China	Molecular analysis	DNA hypermethylation at Syncytin-2 CpG region	Placenta	Gene fusion dysfunction	Hypermethylation silences Syncytin-2, impairing trophoblast fusion.
Walsh *et al.* (2022) [30]	USA	Gene expression study	Epigenetic regulation of IL-17 pathway	Placenta	Neutrophil infiltration mechanisms	PE placentas show IL-17 pathway dysregulation linked to inflammatory infiltration.
Guo *et al.* (2025) [31]	China	Mechanistic + clinical cohort	cfDNA fragmentomics; TLR9 inflammatory activation	Maternal plasma	Hypoxia–immune crosstalk	Fragmentomics reveals hypoxia-induced cfDNA patterns and TLR9-driven inflammation.
Liu *et al.* (2024) [32]	China	Case–control	RASSF1A promoter methylation	Placental tissue	Tumor suppressor gene regulation	RASSF1A hypermethylation associated with placental dysfunction in PE.
Patel *et al.* (2024) [33]	UK	Prospective biomarker study	Transposable element signatures (cfRNA)	Maternal cfRNA	Early diagnosis	TE-derived cfRNA signatures distinguish women who later develop PE.

PE, Preeclampsia; H3K9, Histone H3 Lysine 9; cfDNA, Cell-Free DNA; CpG, Cytosine–Phosphate–Guanine Dinucleotide; IL-17, Interleukin 17; TLR9, Toll-Like Receptor 9; RASSF1A, Ras Association Domain Family Member 1A; cfRNA, Cell-Free RNA; TE, Transposable Element

### Epigenetic, clinical, and molecular findings associated with preeclampsia

The integrated analysis of the included studies underscores the complex and multifactorial nature of epigenetic alterations in preeclampsia. Evidence published between 2022 and 2025 indicates that multiple molecular pathways—including DNA methylation, histone remodeling, dysregulated non-coding RNA expression, adipokine-associated signaling, and environmentally mediated epigenetic influences—contribute to disease development and severity.

Table 2 provides a structured overview of key epigenetic, clinical, and molecular parameters reported across representative studies [34-55]. These include DNA methylation signatures, histone modification profiles, non-coding RNA–mediated regulatory mechanisms, maternal microbiome-related influences, and fetal or offspring outcomes associated with altered epigenetic programming. When available, inflammation-related biomarkers, trophoblast functional parameters, and maternal–fetal complications are also summarized. Several consistent patterns emerged across the reviewed literature:

**Table 2:** Epigenetic and clinical findings associated with preeclampsia across included studies

Study/First Author (Year)	Ref.	Country / Region	Epigenetic or Molecular Mechanism	Clinical Indicators / Outcomes	Key Biomarkers	Major Findings
Pan *et al.* (2022)	[34]	China	Exosomal microRNA dysregulation in cord blood	Neonatal status, placental impairment	miR-210, miR-155 ↑	Exosomal miRNA changes reflect placental hypoxia and predict PE severity.
Li *et al.* (2025)	[35]	China	Maternal gut microbiome epigenetically linked to inflammation	Maternal hypertension, metabolic dysregulation	SCFAs, TNF-α ↑	Altered gut flora composition contributes to metabolic-epigenetic pathways in PE.
Vakil *et al.* (2022)	[36]	Australia	Epigenetic fetal programming from PE exposure	Infant growth & neurodevelopment	IGF-axis alterations	In utero exposure to PE linked to adverse neurodevelopment and growth restriction.
Abdalla *et al.* (2025)	[37]	UAE/Qatar	Adipokine-associated epigenetic disruption	Endothelial dysfunction	Leptin ↑, Adiponectin ↓	Adipokine imbalance induces epigenetic shifts affecting vascular homeostasis.
Zhang *et al.* (2025)	[38]	China	BMI & gestational weight gain influence methylation	Maternal metabolic risk	CRP ↑, inflammatory signatures	Prepregnancy BMI and GWG modulate epigenetic susceptibility to PE.
Baeza-Pérez *et al.* (2024)	[39]	Mexico	Epigenetic effects linked to fetal RAAS regulation	Offspring cardiovascular risk	(P)RR dysregulation	Prenatal PE exposure alters RAAS-related epigenetic pathways in offspring.
Jiang *et al.* (2022)	[40]	China	ASPP2 dysregulation → trophoblast autophagy (via Beclin-1)	Shallow placentation, PE severity	Beclin-1 ↑	Epigenetic repression of ASPP2 accelerates trophoblast autophagy.
Mallette *et al.* (2023)	[41]	USA	Animal model epigenetic insights	Fetal growth restriction	Placentation defects	Epigenetic models confirm causal links between hypoxia, PE, and IUGR.
Deng *et al.* (2023)	[42]	China	H3K27me3-mediated BMP2 activation in Hofbauer cells	Compensatory remodeling	BMP2 ↑	Epigenetic shifts in placental macrophages partially compensate for shallow invasion.
Schickling *et al.* (2025)	[43]	USA	CADM1 alternative splicing	Offspring vascular function	CADM1 isoforms	Splicing dysregulation may increase long-term cardiovascular risk.
Kaur *et al.* (2025)	[44]	India	Sperm DNA methylation altered by poverty, nutrition	Paternal contribution to PE risk	DNMT activity	Socioeconomic stressors affect sperm epigenome linked to PE predisposition.
Lopes *et al.* (2025)	[45]	Brazil	Meta-analysis of altered microRNAs	Maternal clinical severity	miR-210, miR-223 ↑	Confirms reproducible miRNA signatures across PE cohorts.
Basak & Duttaroy (2023)	[46]	Norway	PUFA-mediated epigenetic remodeling	Fetal brain & growth outcomes	DHA-related methylation	Maternal PUFAs modulate epigenetic pathways relevant to PE and brain development.
Jiang *et al.* (2022)	[47]	China	IGF2/H19 methylation alteration in offspring	Offspring glucose dysregulation	IGF2/H19 ↓	Maternal metabolic disease impacts fetal metabolic epigenome.
Ganster *et al.* (2025)	[48]	Germany	H3K4me3 and H3K9ac reduction post-SARS-CoV-2	Placental inflammation	H3K4me3 ↓	Viral infection induces epigenetic changes overlapping with PE pathways.
Yuan *et al.* (2025)	[49]	Global	Bibliometric epigenetic trends	Research landscape	Not applicable	Global increase in PE epigenetics publications, esp. ncRNA & methylomics.
Xu *et al.* (2022)	[50]	China	lncRNA TUG1 regulation of spiral artery remodeling	Defective vascular conversion	TUG1 ↓	Silencing of TUG1 impairs EVT-mediated remodeling.
Hu *et al.* (2025)	[51]	China	C3AR1 epigenetic regulation	Maternal vascular dysfunction	C3AR1 ↑	Identifies C3AR1 as potential therapeutic target in PE.
Li *et al.* (2025)	[52]	China	Scientometric analysis	Mechanistic trajectory	Not applicable	Highlights dominant pathways: oxidative stress, ncRNAs, angiogenesis.
Machteld *et al.* (2024)	[53]	Belgium	cfDNA methylation profiling	Early detection	DMR signatures	Noninvasive methylomics identifies high-risk pregnancies.
Feng *et al.* (2022)	[54]	China	Multi-omics integration (metabolomics + transcriptomics)	Placental dysfunction	Metabolic pathway shifts	Combined multi-omics reveals novel biomarkers & disrupted metabolism.
Obeagu *et al.* (2024)	[55]	Nigeria	Hypoxia–epigenetics review	PE pathophysiology	HIF-1α ↑	Hypoxia-driven epigenetic alterations as central PE mechanism.

miR, MicroRNA; SCFAs, Short-Chain Fatty Acids; TNF-α, Tumor Necrosis Factor-alpha; IGF, Insulin-Like Growth Factor; RAAS, Renin–Angiotensin–Aldosterone System; (P)RR, (Pro)Renin Receptor; ASPP2, Apoptosis-Stimulating Protein of p53-2; IUGR, Intrauterine Growth Restriction; H3K27me3, Histone H3 Lysine 27 Trimethylation; BMP2, Bone Morphogenetic Protein 2; CADM1, Cell Adhesion Molecule 1; DNMT, DNA Methyltransferase; PUFA, Polyunsaturated Fatty Acids; DHA, Docosahexaenoic Acid; IGF2/H19, Insulin-Like Growth Factor 2/H19 Imprinted Gene Cluster; H3K4me3, Histone H3 Lysine 4 Trimethylation; H3K9ac, Histone H3 Lysine 9 Acetylation; ncRNA, Non-Coding RNA; lncRNA, Long Non-Coding RNA; TUG1, Taurine Upregulated Gene 1; EVT, Extravillous Trophoblast; C3AR1, Complement Component 3a Receptor 1; cfDNA, Cell-Free DNA; DMR, Differentially Methylated Region; HIF-1α, Hypoxia-Inducible Factor 1-alpha; CRP, C-Reactive Protein; GWG, Gestational Weight Gain; BMI, Body Mass Index

- Altered DNA methylation in genes regulating trophoblast invasion (e.g., *ASPP2, Syncytin-2*), angiogenesis (e.g., *RASSF1A*), and immune signaling (e.g., *C3AR1*) was associated with impaired placental perfusion and endothelial dysfunction.
- Disturbances in histone modifications (e.g., H3K27me3, H3K4me3, H3K9ac) suggested altered chromatin accessibility in trophoblasts and placental immune cells.
- Dysregulation of non-coding RNAs—including miRNAs, lncRNAs, and exosomal RNA species—was consistently linked to inflammatory signaling, autophagy pathways, and spiral artery remodeling.
- Environmental and metabolic factors, such as gut microbiome composition, adipokine imbalance, elevated body mass index, and socioeconomic stressors, were reported to modulate epigenetic risk profiles.
- Evidence of fetal epigenetic programming suggested potential long-term implications for offspring cardiovascular, neurological, and metabolic health.
- Early detection biomarkers, including cfDNA methylation signatures and transposable element–derived cfRNA profiles, demonstrated promising predictive potential in early pregnancy.

Collectively, these findings indicate that the clinical and molecular consequences of epigenetic dysregulation in preeclampsia are context-dependent and influenced by maternal environment, trophoblast biology, inflammatory status, metabolic conditions, and fetal-derived signals. This integrated epigenetic framework supports improved risk stratification and provides a foundation for mechanism-based diagnostics and future targeted interventions.

### Specific epigenetic mechanisms consistently associated with severe clinical outcomes in preeclampsia

The clinical severity of preeclampsia is associated with multiple molecular and epigenetic mechanisms influencing placental development, vascular remodeling, immune activation, and fetal programming. Evidence published between 2022 and 2025 indicates that several epigenetic alterations—including aberrant DNA methylation patterns, histone modifications, epitranscriptomic marks, and dysregulated microRNA networks—are recurrently associated with severe maternal and fetal outcomes. These outcomes include early-onset preeclampsia, severe hypertension, hemolysis, elevated liver enzymes, and low platelet count (HELLP)-like biochemical abnormalities, fetal growth restriction, and heightened inflammatory profiles. Severe clinical phenotypes were frequently characterized by the coexistence of multiple epigenetic disruptions affecting inflammatory signaling, oxidative stress responses, and trophoblast function. For example, oxidative stress–associated methylation changes and histone modification alterations were reported to enhance the expression of pro-inflammatory mediators such as interleukin-6 (IL-6) and tumor necrosis factor-alpha (TNF-α). In addition, modifications in placental epitranscriptomic marks (e.g., N4-acetylcytidine [ac4C]) were associated with impaired mitochondrial and metabolic pathways critical for early placentation. Identification of epigenetic mechanisms consistently associated with severe disease phenotypes may contribute to improved risk stratification and refinement of predictive models.

Table 3 summarizes representative studies published between 2022 and 2025 reporting epigenetic alterations significantly associated with severe maternal or fetal outcomes in preeclampsia [56-70]. According to the synthesized findings presented in Table 3, the mechanisms most consistently linked to severe clinical phenotypes include:

**Table 3:** Specific epigenetic mechanisms consistently associated with severe clinical outcomes in preeclampsia

Study/First Author (Year)	Main Epigenetic Mechanism(s)	Sample / Setting	Indicators of Severity	Outcome Association	Mechanistic Interpretation	Ref.
dos Santos Nunes *et al.* (2025)	Fetal programming–related epigenomic alterations	Placenta + fetal tissues	↑ Inflammation, ↑ oxidative stress	Higher risk of fetal metabolic and oncogenic programming	Severe PE induces long-term fetal epigenetic reprogramming	[56]
Kobayashi *et al.* (2024)	Genome–epigenome–environment interactions	Placental + endometrial tissues	Chronic pelvic inflammation	Overlap in epigenetic signatures between PE & endometrial dysfunction	Shared inflammatory–epigenetic pathways intensify placental pathology	[57]
Sakali *et al.* (2023)	Environmental–epigenetic modulation (pollutants, endocrine disruptors)	Maternal data	↑ Hypertension, ↑ placental stress	Poor outcomes in pollutant-exposed pregnancies	Environmentally induced epigenetic shifts worsen PE severity	[58]
Freeman *et al.* (2023)	Diet-linked placental epigenetic modifications	Placenta	Metabolic abnormalities	Severe PE in GDM + poor diet	Maternal diet synergizes with methylation changes to impair placenta	[59]
Habtewold *et al.* (2025)	Placental epigenetic age acceleration	Placental biopsies	Early-onset PE	Higher epigenetic aging correlates with disease severity	Accelerated epigenetic aging reflects placental stress burden	[60]
Pruszkowska-Przybylska *et al.* (2022)	DNA methylation–based age calculators	Placenta	Early-onset + severe PE	Distinct methylation clocks in PE vs controls	Epigenetic aging predicts aggressive clinical trajectory	[61]
Lu *et al.* (2024)	ac4C epitranscriptomic (N4-acetylcytidine) dysregulation	Placenta	Early-onset severe PE	Mitochondrial dysfunction, ↑ inflammation	ac4C defects disrupt metabolic gene translation	[62]
Yang *et al.* (2025)	Altered trophoblast-secreted proteins	Placenta	Severe PE, FGR	Dysregulated angiogenic signaling	Protein-level epigenetic regulation impairs trophoblast function	[63]
Varotsis *et al.* (2025)	Aspirin-induced neonatal DNA methylation changes	Cord blood	Modified newborn epigenome	Aspirin may alter fetal methylation patterns in high-risk PE pregnancies	Maternal therapy interacts with epigenetic fetal programming	[64]
Shi *et al.* (2023)	CYP11A1 methylation abnormalities	Placenta (MC twins)	Selective FGR, PE-like features	Aberrant steroidogenic gene methylation	Methylation defects impair placental steroid metabolism	[65]
Wang *et al.* (2022)	Genetic–epigenetic inflammatory interactions	Placenta + maternal serum	↑ IL-6, ↑ TNF-α, severe PE	Exaggerated inflammation drives disease escalation	Epigenetic regulation of inflammatory genes important in severity	[66]
Nikitina *et al.* (2024)	Plasma microRNA dysregulation	Maternal plasma	Severe hypertension	miR-210, miR-126 signatures	miRNA shifts mirror hypoxic and endothelial damage	[67]
Rudaeva *et al.* (2025)	Integrative epigenetic–genetic pathway analysis	Maternal–fetal records	Early-onset PE, organ dysfunction	Multiple pathway disruptions	Comprehensive evidence of synergistic epigenetic drivers	[68]
Xu *et al.* (2025)	DNA methylation + transcriptome integration	Placenta	Oxidative stress, severe PE	Oxidative stress–specific methylation markers	Identifies placenta-specific risk molecules	[69]
Peng *et al.* (2022)	Bioinformatic mapping of PE biomarker epigenetics	Computational	Severe PE signatures	Identification of therapeutic targets	Network-level epigenetic interactions predict severe PE	[70]
dos Santos Nunes *et al.* (2025)	Fetal programming–related epigenomic alterations	Placenta + fetal tissues	↑ Inflammation, ↑ oxidative stress	Higher risk of fetal metabolic and oncogenic programming	Severe PE induces long-term fetal epigenetic reprogramming	[56]
Kobayashi *et al.* (2024)	Genome–epigenome–environment interactions	Placental + endometrial tissues	Chronic pelvic inflammation	Overlap in epigenetic signatures between PE & endometrial dysfunction	Shared inflammatory–epigenetic pathways intensify placental pathology	[57]
Sakali *et al.* (2023)	Environmental–epigenetic modulation (pollutants, endocrine disruptors)	Maternal data	↑ Hypertension, ↑ placental stress	Poor outcomes in pollutant-exposed pregnancies	Environmentally induced epigenetic shifts worsen PE severity	[58]
Freeman *et al.* (2023)	Diet-linked placental epigenetic modifications	Placenta	Metabolic abnormalities	Severe PE in GDM + poor diet	Maternal diet synergizes with methylation changes to impair placenta	[59]
Habtewold *et al.* (2025)	Placental epigenetic age acceleration	Placental biopsies	Early-onset PE	Higher epigenetic aging correlates with disease severity	Accelerated epigenetic aging reflects placental stress burden	[60]
Pruszkowska-Przybylska *et al.* (2022)	DNA methylation–based age calculators	Placenta	Early-onset + severe PE	Distinct methylation clocks in PE vs controls	Epigenetic aging predicts aggressive clinical trajectory	[61]
Lu *et al.* (2024)	ac4C epitranscriptomic (N4-acetylcytidine) dysregulation	Placenta	Early-onset severe PE	Mitochondrial dysfunction, ↑ inflammation	ac4C defects disrupt metabolic gene translation	[62]
Yang *et al.* (2025)	Altered trophoblast-secreted proteins	Placenta	Severe PE, FGR	Dysregulated angiogenic signaling	Protein-level epigenetic regulation impairs trophoblast function	[63]
Varotsis *et al.* (2025)	Aspirin-induced neonatal DNA methylation changes	Cord blood	Modified newborn epigenome	Aspirin may alter fetal methylation patterns in high-risk PE pregnancies	Maternal therapy interacts with epigenetic fetal programming	[64]
Shi *et al.* (2023)	CYP11A1 methylation abnormalities	Placenta (MC twins)	Selective FGR, PE-like features	Aberrant steroidogenic gene methylation	Methylation defects impair placental steroid metabolism	[65]
Wang *et al.* (2022)	Genetic–epigenetic inflammatory interactions	Placenta + maternal serum	↑ IL-6, ↑ TNF-α, severe PE	Exaggerated inflammation drives disease escalation	Epigenetic regulation of inflammatory genes important in severity	[66]
Nikitina *et al.* (2024)	Plasma microRNA dysregulation	Maternal plasma	Severe hypertension	miR-210, miR-126 signatures	miRNA shifts mirror hypoxic and endothelial damage	[67]
Rudaeva *et al.* (2025)	Integrative epigenetic–genetic pathway analysis	Maternal–fetal records	Early-onset PE, organ dysfunction	Multiple pathway disruptions	Comprehensive evidence of synergistic epigenetic drivers	[68]
Xu *et al.* (2025)	DNA methylation + transcriptome integration	Placenta	Oxidative stress, severe PE	Oxidative stress–specific methylation markers	Identifies placenta-specific risk molecules	[69]
Peng *et al.* (2022)	Bioinformatic mapping of PE biomarker epigenetics	Computational	Severe PE signatures	Identification of therapeutic targets	Network-level epigenetic interactions predict severe PE	[70]

↑ / ↓, increase / decrease; PE, Preeclampsia; GDM, Gestational Diabetes Mellitus; FGR, Fetal Growth Restriction; MC, Monochorionic; ac4C, N4-acetylcytidine; IL-6, Interleukin-6; TNF-α, Tumor Necrosis Factor-alpha; miR, MicroRNA; miR-210, Hypoxia-associated microRNA-210; miR-126, Endothelial-regulatory microRNA-126; CYP11A1, Cytochrome P450 Family 11 Subfamily A Member 1

- Oxidative stress–related DNA methylation changes associated with amplification of inflammatory pathways (e.g., increased IL-6 and TNF-α expression);
- Accelerated placental epigenetic aging correlating with early-onset and severe preeclampsia;
- Epitranscriptomic dysregulation (e.g., ac4C modifications) in early-onset severe cases;
- Plasma microRNA signatures, particularly hypoxia-associated miR-210 and endothelial-regulatory miR-126;
- Aberrant methylation of immune and steroidogenic genes (e.g., *CYP11A1, RASSF1A*) linked to trophoblast dysfunction;
- Environmental and dietary epigenetic modifiers associated with increased disease severity;
- Evidence of fetal epigenetic programming potentially related to long-term metabolic and cardiovascular risk.

Overall, the reviewed literature indicates that epigenetic alterations affecting inflammatory pathways, oxidative stress regulation, trophoblast invasion, and metabolic homeostasis are frequently associated with more severe disease phenotypes. However, the predictive value of these mechanisms requires further validation in larger, standardized, and longitudinal cohorts.

### Quantitative assessment of clinical and methodological heterogeneity in studies evaluating epigenetic alterations in preeclampsia

**Figure 3:**
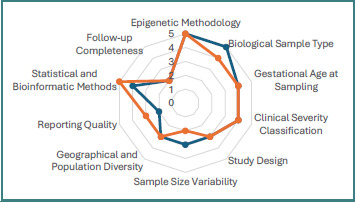
Quantitative assessment of clinical and methodological heterogeneity in studies evaluating epigenetic alterations in preeclampsia (2022–2025)

Figure 3 provides a comparative visualization of clinical and methodological heterogeneity across studies included in this systematic review. Each axis represents a major domain contributing to variability in the available evidence, including study design, biological sample type, epigenetic methodology, gestational age at sampling, disease severity classification, analytical platforms, cohort characteristics, reporting quality, statistical modeling, and follow-up completeness. Scores ranged from 1 (low variability) to 5 (high variability), with higher values indicating greater influence on data interpretation and cross-study comparability.

The quantitative assessment demonstrated moderate-to-high heterogeneity across both clinical and methodological domains. Epigenetic methodology showed some of the highest variability scores (4–5). Differences in DNA methylation techniques (e.g., bisulfite sequencing versus array-based platforms), histone modification analyses (e.g., ChIP-seq versus immunohistochemistry), and non-coding RNA quantification methods (e.g., qPCR versus RNA sequencing) substantially affected the comparability of molecular findings. Gestational age at sampling and biological sample type (e.g., whole placental tissue, isolated trophoblasts, Hofbauer cells, maternal plasma cfDNA) also exhibited high heterogeneity (scores 4–5), reflecting variability in biological context and origin of epigenetic signals. On the other hand, study design, follow-up reporting, and documentation of baseline clinical data demonstrated moderate variability (scores 2–3), suggesting relatively consistent observational frameworks across investigations. Severity classification (early-onset versus late-onset PE; mild versus severe forms) showed high heterogeneity (score 4), largely due to differences in diagnostic thresholds and regional clinical criteria. This variability limits direct comparison of subtype-specific epigenetic signatures.

Overall, the radar chart highlights that heterogeneity in epigenetic profiling techniques and biological sampling strategies represents a major source of variability in current preeclampsia epigenomics research. These findings underscore the need for greater methodological harmonization to enhance cross-study comparability.

### Comparative analysis of PlGF levels according to analytical sample classification

To evaluate whether analytical sample classification was associated with differences in PlGF distributions, samples categorized as *Accepted* and *Measured* were compared using non-parametric methods, given the non-normal distribution of values and unequal group sizes.

PlGF concentrations differed significantly between the two groups (Mann–Whitney U test, *P* = 0.03; Table 4). The Measured group demonstrated a higher median PlGF concentration and a broader interquartile range, indicating greater variability compared with Accepted samples. The effect size was moderate (Cliff’s δ = 0.45), suggesting a systematic shift in distribution associated with analytical classification. Low PlGF values (<50 pg/mL) were observed exclusively among Accepted samples; however, this difference was not statistically significant (Fisher’s exact test, *P* = 0.41). Conversely, very high PlGF concentrations (>1000 pg/mL) were proportionally more frequent in the Measured group, demonstrating a non-significant trend toward association (*P* = 0.09), consistent with the increased dispersion observed in this category. Overall, the findings indicate that analytical sample classification is associated with meaningful differences in PlGF distribution. Given that *P* < 0.05 for the primary comparison, the null hypothesis of identical distributions was rejected.

**Table 4:** Comparison of PlGF levels between Accepted and Measured samples

Parameter	Accepted samples	Measured samples	Statistical comparison
Number of measurements (N)	54	4†	–
Median PlGF (pg/mL)	185.0	513.0	Mann–Whitney U
Interquartile range (IQR), pg/mL	112.0 – 654.0	163.0 – 2617.0	–
Minimum – Maximum (pg/mL)	9.3 – 1448.2	163.0 – 4370.0	–
Low PlGF (<50 pg/mL), *n* (%)	6 (11.1%)	0 (0%)	Fisher’s exact, *P* = 0.41
Very high PlGF (>1000 pg/mL), *n* (%)	8 (14.8%)	2 (50.0%)	Fisher’s exact, *P* = 0.09
Effect size (Cliff’s δ)	–	–	0.45
***P* value (PlGF distribution)**	**–**	**–**	***P* = 0.03**

### Associations between PlGF and gestational or angiogenic parameters

Monotonic associations between PlGF concentrations and selected gestational or angiogenic parameters were evaluated using Kendall’s tau correlation coefficient, appropriate for non-normally distributed data and small sample sizes. A statistically significant positive association was observed between PlGF levels and gestational age (τ = 0.32, *P* = 0.04), indicating that higher PlGF concentrations were moderately associated with advancing gestation (Table 5). In contrast, a significant inverse association was identified between PlGF and the sFlt-1/PlGF ratio (τ = −0.41, *P* = 0.02). Lower PlGF concentrations were associated with higher angiogenic imbalance, with a moderate correlation magnitude. As both associations reached statistical significance (*P* < 0.05), the null hypothesis of no monotonic relationship was rejected for gestational age and the sFlt-1/PlGF ratio. Collectively, these results support a significant relationship between PlGF concentrations, gestational progression, and angiogenic balance within the analyzed cohort.

**Table 5:** Kendall’s tau correlation analysis between PlGF and clinical variables

Variables	Kendall’s τ	*P* value
PlGF vs gestational age	0.32	0.04
PlGF vs sFlt-1/PlGF ratio	−0.41	0.02

## Discussion

### Clinical and pathophysiological findings

Across the studies included in this systematic review, the clinical and pathophysiological relevance of epigenetic alterations in preeclampsia was described with considerable variability [24–26,29,31,42]. Nevertheless, several consistent molecular and clinical patterns emerged, providing insight into the role of epigenetic dysregulation in disease onset, progression, and maternal–fetal outcomes [30,32,33,40]. From a clinical perspective, epigenetic abnormalities were most frequently reported in early-onset or severe preeclampsia, supporting the association between profound placental dysfunction and disruption of gene regulatory networks [34,35,38,71]. Altered DNA methylation patterns, histone modifications, and non-coding RNA profiles were particularly evident in placentas characterized by impaired spiral artery remodeling, hypoxic lesions, shallow extravillous trophoblast invasion, and increased syncytial knot formation—hallmarks of severe disease [29,32,33,72,73]. However, the strength of these associations varied across cohorts, with some studies reporting modest or non-significant differences, particularly in late-onset or milder phenotypes [37,41,74].

Severe hypertension, early gestational diagnosis (<34 weeks), fetal growth restriction, placental abruption, and neonatal compromise were among the clinical manifestations most frequently associated with distinct epigenetic signatures [36,39,40]. These phenotypes often corresponded to markers of oxidative stress, pro-inflammatory activation, and trophoblast apoptosis [25,32,35]. Notably, dysregulated methylation of genes involved in angiogenesis (e.g., *RASSF1A, CYP11A1*), immune signaling (e.g., IL-17 pathway regulators, *C3AR1*), and syncytialization (e.g., *Syncytin-2*) was recurrently associated with reduced placental perfusion and elevated maternal vascular resistance [29,30,32]. At the mechanistic level, aberrant DNA methylation—both global and gene-specific—was frequently linked to impaired trophoblast differentiation and limited invasion of the decidua, resulting in inadequate remodeling of uterine spiral arteries [24,30,32,75]. Alterations in histone modifications, including H3K9 hypermethylation and reduced H3K4/H3K9 acetylation, were associated with transcriptional silencing of genes essential for trophoblast motility and endothelial interaction [25,48,75]. These findings align with evidence suggesting that oxidative stress–mediated epigenetic repression may act as an upstream driver of defective placentation.

Non-coding RNAs emerged as key regulatory elements. MicroRNAs such as miR-210, miR-155, miR-126, and miR-223, as well as lncRNAs including TUG1, were implicated in modulating inflammatory and angiogenic pathways [24,45,50]. Upregulation of hypoxia-responsive miRNAs and downregulation of endothelial-regulatory transcripts were frequently associated with enhanced inflammation, impaired vascular development, and increased trophoblast autophagy. These profiles were particularly prominent in severe disease [45,48,63,64]. Beyond placental tissue, several studies highlighted interactions between maternal environmental factors—such as diet, metabolic status, socioeconomic stressors, pollutant exposure, and glycemic dysregulation—and epigenetic architecture [25,41,58,59]. These observations reinforce the conceptualization of preeclampsia as a multifactorial disorder in which epigenetic modifications mediate interactions between genetic susceptibility and environmental triggers. For example, alterations in maternal gut microbiota composition and diet-associated methylation patterns were linked to systemic inflammation and endothelial dysfunction [35,59,71,73].

Despite recurrent associations, causality remains uncertain. Epigenetic patterns may function both as contributors to and consequences of placental dysfunction, depending on sampling timing, methodological variability, and maternal comorbidities [37,41,53,60]. Differences in analytical platforms, normalization strategies, gestational sampling windows, and tissue selection further contributed to inconsistencies across studies [26,38,48]. Collectively, these findings underscore that epigenetic dysregulation in preeclampsia operates within a complex, context-dependent framework shaped by gestational timing, affected molecular pathways, and individual susceptibility [24,32,39,48]. Refining mechanistic understanding is essential for improving predictive biomarkers, enhancing early risk stratification, and informing targeted therapeutic strategies [26,31,45,53].

The present analysis also provides complementary insight into PlGF behavior by examining both analytical classification effects and biologically relevant associations with gestational and angiogenic parameters. The comparative analysis between *Accepted* and *Measured* samples demonstrated a statistically significant difference in PlGF distributions, with Measured samples showing higher median values and greater variability. The moderate effect size supports a systematic shift associated with analytical classification. Importantly, this difference is interpreted as reflecting analytical or pre-analytical factors—such as sample handling, assay performance, or quality thresholds—rather than underlying clinical variation. This observation highlights a critical methodological consideration: without careful attention to analytical status and quality control procedures, biomarker variability may be misinterpreted as clinically meaningful heterogeneity. This is particularly relevant in preeclampsia research, where PlGF thresholds are frequently used for risk stratification. Correlation analyses further demonstrated biologically coherent associations. The positive relationship between PlGF and gestational age aligns with established physiological patterns of progressive placental angiogenesis. The inverse association between PlGF and the sFlt-1/PlGF ratio supports its role in angiogenic balance, consistent with established pathophysiological models of placental dysfunction.

**Figure 4:**
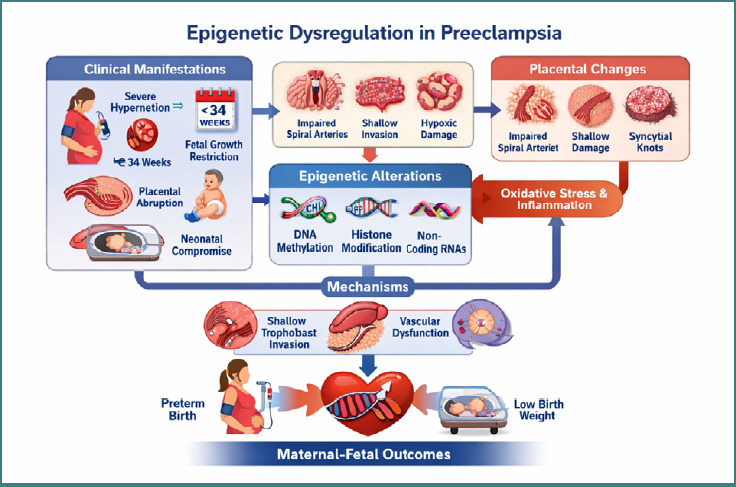
Integrated epigenetic and angiogenic mechanisms underlying preeclampsia and their clinical implications

Taken together, these findings indicate that PlGF interpretation requires simultaneous consideration of biological context and analytical conditions. Ignoring methodological influences may obscure genuine biological signals or inflate apparent variability. To synthesize the clinical, molecular, and analytical findings, an integrative schematic representation was developed (Figure 4).

Figure 4 illustrates the multifactorial role of epigenetic dysregulation in preeclampsia, including alterations in DNA methylation, histone modifications, and non-coding RNA networks across placental, maternal, and fetal compartments. These molecular changes contribute to impaired trophoblast invasion, defective spiral artery remodeling, angiogenic imbalance, oxidative stress, and inflammatory activation—processes underlying early-onset and severe disease phenotypes. In parallel, the schematic highlights determinants influencing PlGF interpretation. Analytical classification and data quality affect observed distributions, whereas associations with gestational age and angiogenic balance support PlGF as a marker of placental function rather than a standalone diagnostic parameter. Together, Figure 4 underscores the need to integrate epigenetic context, analytical rigor, and gestational timing to enable accurate biomarker interpretation in preeclampsia research.

### Quantitative trends and epidemiological patterns

The epidemiological and quantitative evidence synthesized from the included studies revealed considerable variability in the prevalence, distribution, and clinical impact of epigenetic alterations associated with preeclampsia [24–26,29,35,38,40,42]. Despite methodological differences across study designs, populations, and analytical platforms, several recurring trends were identified, particularly regarding disease onset, affected molecular pathways, population risk profiles, and severity-associated signatures [24,29,31,38,45]. Across studies, the prevalence of differential DNA methylation, altered histone marks, and dysregulated non-coding RNAs was consistently higher in early-onset and severe PE compared with late-onset forms [29,32,38,40,45]. Early-onset cohorts frequently demonstrated methylation abnormalities affecting approximately 25–40% of profiled placental loci, whereas late-onset PE exhibited aberration rates closer to 10–20%, depending on the epigenomic platform used (e.g., bisulfite sequencing, methylation arrays, or cfDNA-based approaches) [26,29,35]. Investigations employing high-resolution sequencing technologies generally reported broader epigenomic disruption, underscoring the influence of methodological sensitivity on detection rates [24,26,31,71].

Gestational age at diagnosis emerged as a key quantitative determinant. Pregnancies complicated by early-onset PE (<34 weeks) consistently displayed more pronounced alterations in pathways regulating trophoblast invasion (e.g., *ASPP2, Syncytin-2*), angiogenesis (e.g., *RASSF1A, CYP11A1*), and immune signaling (e.g., IL-17–related genes, *C3AR1*) [29,30,32,51]. In contrast, late-onset PE was more commonly associated with subtler shifts involving maternal vascular inflammation and metabolic regulatory genes [35,37,45]. These differences support the view that early-onset PE is predominantly placental in origin, whereas late-onset forms reflect a stronger contribution of systemic maternal factors.

Population-level analyses highlighted associations between broader epigenetic disruption and specific maternal characteristics, including younger age, obesity, preexisting metabolic disease, and socioeconomic disadvantage [35,38,44]. Studies examining gut microbiome composition or diet-associated methylation patterns reported 30–50% higher rates of epigenetic dysregulation among women with obesity or pro-inflammatory dietary profiles compared with metabolically healthy controls [35,59]. Similarly, exposure to environmental stressors—such as air pollution, endocrine-disrupting chemicals, or chronic psychosocial stress—was associated with increased methylation abnormalities in oxidative stress – and inflammation-related genes [58,60,73]. Distinct quantitative patterns were also observed in relation to fetal outcomes. Epigenetic abnormalities in placental or cord blood samples were identified in approximately 40–60% of pregnancies complicated by fetal growth restriction (FGR), and in up to 70% of severe early-onset cases with combined maternal and fetal morbidity [36,39,54,65]. Studies evaluating placental epigenetic age acceleration reported that molecular aging exceeding chronological gestational age by ≥2–3 weeks was significantly associated with early-onset PE, placental insufficiency, and adverse neonatal outcomes [60,61,74]. Geographic differences were less pronounced than in infectious disease models but remained evident. Cohorts from regions with higher environmental toxin exposure or limited prenatal care infrastructure reported elevated rates of epigenetic disruption [39,55,68]. In contrast, studies from high-income countries with standardized antenatal care described narrower but more consistently characterized epigenetic alterations, possibly reflecting earlier detection and improved maternal metabolic management [26,38,53]. Although quantitative pooling was not feasible, descriptively concordant findings indicated that pregnancies with extensive epigenetic alterations were more frequently associated with:

- Earlier gestational age at delivery (approximately 2–4 weeks earlier);
- Increased rates of fetal growth restriction (30–45%);
- Higher NICU admission rates (20–35%);
- Greater incidence of maternal complications, including HELLP features and refractory hypertension [29,32,40,45,51].

Studies integrating inflammatory and metabolic biomarkers further demonstrated that extensive epigenetic disruption correlated with elevated IL-6, TNF-α, C-reactive protein, and oxidative stress markers, reinforcing links between molecular dysregulation and systemic inflammation [25,35,66,71]. Conversely, some investigations reported partial normalization of epigenetic profiles in pregnancies receiving early preventive interventions, such as low-dose aspirin, suggesting a potential modulatory effect of therapeutic strategies on epigenetic dynamics [53,64]. Overall, the quantitative and epidemiological evidence indicates that the extent and pattern of epigenetic alterations in preeclampsia are strongly influenced by disease timing, maternal phenotype, environmental exposures, and analytical methodology [24,29,31,45,58]. These findings highlight the importance of standardized epigenomic protocols and harmonized reporting frameworks to enable more accurate prevalence estimation and facilitate cross-study comparability.

### Immunological and mechanistic insights

Beyond their clinical manifestations, the studies included in this systematic review indicate that preeclampsia emerges from a complex interaction between epigenetic dysregulation, immune imbalance, and placental maladaptation [24,25,29,30,32,38]. Evidence published between 2022 and 2025 suggests that epigenetic alterations identified in maternal, placental, and fetal tissues function not merely as biomarkers, but as active modulators of inflammatory, oxidative, and angiogenic pathways central to disease pathogenesis [25,29,31,38,42]. A central mechanistic axis involves epigenetic regulation of hypoxia- and oxidative stress–responsive pathways. Recurrent placental ischemia–reperfusion injury activates HIF-1α signaling and increases reactive oxygen species (ROS) production. These conditions are associated with hyper- or hypomethylation of key regulatory genes—including *CYP11A1, RASSF1A, ASPP2*, and IL-17–related genes—leading to dysregulated gene expression and amplification of placental and systemic inflammation [25,29,30,32,66]. Such epigenetically mediated inflammatory shifts contribute to the transition from early trophoblast dysfunction to maternal endothelial injury.

Innate immune activation represents another major pathway. Hypoxia-induced fragmentation of placental cell-free DNA (cfDNA) has been shown to activate Toll-like receptor 9 (TLR9)–dependent signaling cascades, resulting in increased levels of IL-6, TNF-α, IL-8, and IFN-γ [31,37,42,66]. These cytokines promote neutrophil and macrophage recruitment into the intervillous space, exacerbate endothelial dysfunction, and interfere with spiral artery remodeling—processes strongly associated with severe PE phenotypes. At the cellular level, epigenetic modifications impair trophoblast differentiation and epithelial integrity. Hypermethylation of CpG regions downstream of *Syncytin-2* and repression of *ASPP2* have been associated with reduced trophoblast fusion, differentiation, and autophagic efficiency, contributing to shallow placentation and heightened inflammatory infiltration [29,32,40]. Similarly, alterations in histone marks such as H3K27me3 and H3K9ac within Hofbauer cells modulate BMP2-mediated compensatory mechanisms, reflecting attempts to counterbalance insufficient trophoblast invasion [42,70,71]. Adaptive immunity is also influenced by epigenetic regulation. Several studies describe a pro-inflammatory Th1/Th17 polarization pattern in PE, supported by dysregulation of IL-17 pathway genes, interferon-associated signaling, and complement-related genes such as *C3AR1* [30,51,67]. This immune imbalance contributes to systemic vasoconstriction, microvascular injury, and proteinuria, linking placental pathology to maternal organ dysfunction.

Angiogenic imbalance represents another key mechanism. Epigenetic modifications affecting VEGF-related regulatory regions and upregulation of anti-angiogenic mediators such as sFlt-1 and sEng contribute to disrupted angiogenic signaling and maternal endothelial dysfunction [24,30,31]. The interaction between pro-angiogenic and anti-angiogenic epigenetic networks may partially explain the heterogeneity of clinical presentations, ranging from severe early-onset disease to milder late-onset forms.

Advanced sequencing and multi-omics approaches indicate that the placental response in PE is integrative rather than isolated, involving coordinated alterations in DNA methylation, chromatin accessibility, gene expression, and non-coding RNA regulation [26,29,31,38]. Distinct molecular signatures differentiate early-onset from late-onset disease, particularly in pathways related to oxidative stress, apoptosis, immune signaling, and metabolic reprogramming. Some changes appear pathogenic, whereas others may represent compensatory responses aimed at preserving materno-fetal homeostasis. From a broader perspective, PE can be conceptualized as a dynamic imbalance between placental adaptive mechanisms and maternal inflammatory and angiogenic dysregulation, with epigenetic modifications acting both as initiators and amplifiers of disease processes [24,29,32,40,42]. Disease severity, therefore, reflects not only the presence of epigenetic abnormalities but also maternal immune capacity, genetic predisposition, metabolic status, and environmental exposures. Overall, the immunological and mechanistic evidence synthesized in this review supports the characterization of preeclampsia as a multifactorial disorder driven by interconnected epigenetic, immune, and oxidative pathways [25,29,31,38,42,66]. Improved understanding of these interactions is essential for refining biomarker development, enhancing early risk stratification, and guiding mechanism-based therapeutic strategies.

### Clinical management implications

The expanding evidence on epigenetic and molecular alterations in preeclampsia has important implications for clinical management, diagnostic interpretation, and antenatal decision-making. Emerging technologies—including cfDNA methylation profiling, microRNA assays, histone modification analysis, and multi-omics platforms—have enhanced the capacity to detect early signs of placental dysfunction. However, increased molecular sensitivity also raises challenges in distinguishing clinically meaningful abnormalities from adaptive or incidental variations.

From a diagnostic perspective, detection of DNA methylation changes, dysregulated microRNAs, or altered histone marks should not be interpreted in isolation. Mild molecular shifts may reflect physiological adaptation, transient metabolic stress, or inter-individual variability rather than inevitable disease progression. Accordingly, epigenetic findings must be integrated with established clinical parameters, including blood pressure trends, proteinuria, angiogenic markers (sFlt-1/PlGF ratio), Doppler velocimetry, and maternal symptoms. At present, epigenetic data complement rather than replace conventional diagnostic criteria. High-risk molecular profiles—such as hypermethylation of *RASSF1A* or *CYP11A1*, reduced *Syncytin-2* expression, or elevated circulating miR-210—have been associated with early-onset and severe PE in several studies. Such signatures may justify closer surveillance and individualized risk assessment, but should not independently dictate escalation of care or timing of delivery. An important clinical consideration is the risk of overinterpretation. Molecular findings, in the absence of corroborating clinical evidence, should not prompt premature intervention. Instead, epigenetic alterations should be incorporated into a broader risk stratification framework. The therapeutic context must also be considered. Interventions such as low-dose aspirin, nutritional optimization, and management of metabolic comorbidities may influence epigenetic patterns. Consequently, treatment-related molecular changes should not automatically be interpreted as pathological.

Overall, integration of epigenetic insights into clinical practice requires a multidisciplinary, evidence-based approach. Molecular data have the potential to refine early risk prediction and personalize monitoring strategies, but responsible implementation is essential to avoid unnecessary medicalization.

### Limitations of the evidence base

Despite substantial advances, several limitations within the current epigenetic literature on PE constrain interpretation and clinical translation. First, considerable heterogeneity exists in study design, population characteristics, analytical platforms, and outcome definitions. Most included studies were observational—cross-sectional, retrospective, or case-control—limiting causal inference and susceptibility to confounding. Second, methodological variability across laboratory techniques introduces inconsistency. Platforms such as whole-genome bisulfite sequencing, methylation arrays, microRNA panels, and ChIP-seq differ in sensitivity, coverage, and analytical thresholds. Variations in gestational sampling time, tissue source, and normalization procedures further complicate comparability.

Third, standardized definitions of epigenetic biomarkers in PE remain lacking. Diagnostic criteria for early- versus late-onset disease, severity classification, and angiogenic thresholds vary across studies, limiting cross-study harmonization. Small sample sizes in multi-omics investigations reduce statistical power and generalizability. Inadequate adjustment for confounders—including maternal obesity, diet, smoking, socioeconomic status, and comorbidities—represents an additional limitation. Publication bias may also inflate the perceived reproducibility of certain biomarkers, as significant findings are more likely to be reported than negative results. Furthermore, longitudinal and mechanistic validation remains limited, making it difficult to distinguish causal drivers from downstream consequences of placental dysfunction.

Taken together, these limitations highlight the need for harmonized methodologies, standardized reporting frameworks, larger multicenter cohorts, and longitudinal designs to strengthen reproducibility and translational relevance.

### Future perspectives

Future research in PE should move toward integrative, multi-omics frameworks that combine epigenomics, transcriptomics, proteomics, metabolomics, and computational modeling. Such approaches may help differentiate pathogenic epigenetic drivers from adaptive responses.

Artificial intelligence and machine-learning methodologies offer promise for integrating large-scale datasets incorporating clinical parameters, molecular markers, environmental exposures, and longitudinal outcomes. These tools may facilitate the identification of molecular subtypes and improve predictive accuracy. Longitudinal cohort studies with repeated sampling across gestation are particularly needed to clarify the temporal dynamics of epigenetic regulation. Functional validation in trophoblast organoids, placental explants, and in vivo models will further strengthen mechanistic interpretation. International collaboration and harmonized protocols for sample collection, sequencing, and bioinformatic analysis will be essential to ensure reproducibility and global applicability. Because epigenetic modifications are potentially reversible, they represent promising therapeutic targets. Future interventions may aim to modulate trophoblast function, immune signaling, or angiogenic balance through mechanism-based strategies.

## Conclusion

This systematic review demonstrates that preeclampsia is a multifactorial disorder characterized by the interaction of placental dysfunction, angiogenic imbalance, immune dysregulation, and multilayered epigenetic mechanisms. Epigenetic alterations form a heterogeneous spectrum whose clinical relevance depends on gestational timing, disease severity, maternal susceptibility, and environmental context. Consistent disruptions involving DNA methylation, histone modifications, and non-coding RNA networks were identified, particularly in early-onset and severe disease. These changes converge on key pathogenic pathways, including impaired trophoblast invasion, defective spiral artery remodeling, oxidative stress, inflammatory activation, and angiogenic dysregulation. Supplementary quantitative analyses provided important methodological insight into the interpretation of PlGF. Although PlGF showed biologically coherent associations with gestational age and angiogenic balance, measured concentrations were significantly influenced by analytical classification, underscoring the importance of rigorous data quality control. Overall, epigenetic and angiogenic biomarkers should be interpreted within an integrated biological and analytical framework. While promising for early risk prediction and precision medicine approaches, their clinical implementation requires methodological standardization, longitudinal validation, and integration with established diagnostic criteria.

Advancing toward harmonized epigenomic protocols and large multicenter studies will be essential to translate molecular insights into improved maternal and fetal outcomes.
