# Supplementary material for: Epigenetic alterations in preeclampsia: a systematic review of current mechanisms and biomarker potential
Source: J Med Life. 2026 Feb;19(2):69–88. doi: 10.25122/jml-2026-0009 (PMC13059462; doi:10.25122/jml-2026-0009)
Supplement: Supplementary file 1 [file JMedLife-19-069-s001.pdf]

Table A1. Database search strategy

Database	Date of Last Search	Search Terms (MeSH + Keywords)	Boolean Logic / Query Structure	Filters Applied
PubMed	30 September 2025	preeclampsia, pre-eclampsia, hypertensive disorders of pregnancy, placenta, placental dysfunction, epigenetics, DNA methylation, histone modification, microRNA, miRNA, lncRNA, non-coding RNA, epigenetic biomarker, trophoblast, angiogenesis, oxidative stress	("preeclampsia"[MeSH] OR "pre-eclampsia" OR "hypertensive disorders of pregnancy") AND ("epigenomics" OR "epigenetics" OR "DNA methylation" OR "histone modification" OR "microRNA" OR "non-coding RNA") AND ("placenta"[MeSH] OR "trophoblast" OR "placental dysfunction")	Humans; English; Publication date: 1 Jan 2022–30 Sept 2025
Web of Science	30 September 2025	preeclampsia, placental epigenetics, DNA methylation, histone acetylation, chromatin remodeling, miRNA, lncRNA, epigenetic regulation, placental gene expression	TS = (preeclampsia OR "hypertensive pregnancy disorder") AND TS = ("DNA methylation" OR "histone modification" OR microRNA OR lncRNA OR "epigenetic marker") AND TS = (placenta OR trophoblast OR "placental development")	Document type: Article; Language: English; Timespan: 2022–2025

© 2026 by the authors. This article is an open access article distributed under the terms and conditions of the Creative Commons Attribution (CC BY 4.0) license.
